# 5-Amino-3-anilino-1*H*-pyrazole-4-carbonitrile

**DOI:** 10.1107/S1600536812036045

**Published:** 2012-08-25

**Authors:** Shaaban K. Mohamed, Mehmet Akkurt, Frank R. Fronczek, Mahmoud A. A. El-Remaily, Antar A. Abdelhamid

**Affiliations:** a Chemistry and Environmental Division, Manchester Metropolitan University, Manchester M1 5GD, England; b Department of Physics, Faculty of Sciences, Erciyes University, 38039 Kayseri, Turkey; c Department of Chemistry, Louisiana State University, Baton Rouge, LA 70803-1804, USA; d Department of Chemistry, Faculty of Science, Sohag University, 82524 Sohag, Egypt

## Abstract

In the title compound, C_10_H_9_N_5_, the phenyl ring is twisted with respect to the pyrazole ring, forming a dihedral angle of 24.00 (6)°. In the crystal, mol­ecules are linked by N—H⋯N hydrogen bonds into chains running parallel to [010] containing alternating *R*_2_^2^(6) and *R*_2_^2^(12) rings. Further inter­actions are found in the crystal, *viz*. N—H⋯π(phen­yl) inter­actions and weak face-to-face π–π stacking inter­actions [centroid–centroid distance = 3.8890 (6) Å] between the centroids of the pyrazole and phenyl rings are observed.

## Related literature

For biological activities of pyrazoles, see: Kaushik *et al.* (2010 ▶); Sheikh *et al.* (2009 ▶); Krishnamurthy *et al.* (2004 ▶); Grimmett (1970 ▶). For the use of related compounds as bridging ligands, see: Lynch & McClenaghan (2005 ▶). For the synthesis of the title compound, see: Soliman *et al.* (2010 ▶). For hydrogen-bond motifs, see: Bernstein *et al.* (1995 ▶).
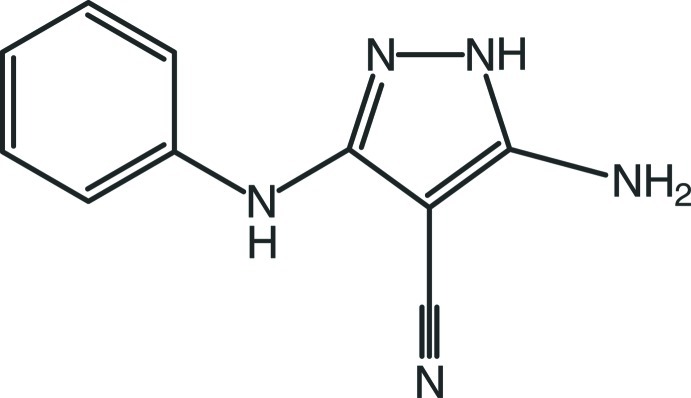


## Experimental

### 

#### Crystal data

C_10_H_9_N_5_
*M* *_r_* = 199.22
Orthorhombic, 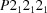

*a* = 6.3441 (1) Å
*b* = 11.1354 (2) Å
*c* = 13.7754 (3) Å
*V* = 973.15 (3) Å^3^
*Z* = 4
Mo *K*α radiation
μ = 0.09 mm^−1^
*T* = 90 K
0.25 × 0.17 × 0.08 mm

#### Data collection

Bruker Kappa APEXII DUO diffractometer
Absorption correction: multi-scan (*SADABS*; Sheldrick, 2004 ▶) *T*_min_ = 0.978, *T*_max_ = 0.993
32878 measured reflections
2975 independent reflections
2767 reflections with *I* > 2σ(*I*)
*R*_int_ = 0.035
Standard reflections: 0

#### Refinement

*R*[*F* ^2^ > 2σ(*F* ^2^)] = 0.033
*wR*(*F* ^2^) = 0.086
*S* = 1.09
2975 reflections
152 parameters
4 restraints
H atoms treated by a mixture of independent and constrained refinement
Δρ_max_ = 0.32 e Å^−3^
Δρ_min_ = −0.20 e Å^−3^

### 

Data collection: *APEX2* (Bruker, 2007 ▶); cell refinement: *SAINT* (Bruker, 2007 ▶); data reduction: *SAINT*; program(s) used to solve structure: *SIR97* (Altomare *et al.*, 1999 ▶); program(s) used to refine structure: *SHELXL97* (Sheldrick, 2008 ▶); molecular graphics: *ORTEP-3 for Windows* (Farrugia, 1997 ▶) and *PLATON* (Spek, 2009 ▶); software used to prepare material for publication: *WinGX* (Farrugia, 1999 ▶) and *PLATON*.

## Supplementary Material

Crystal structure: contains datablock(s) global, I. DOI: 10.1107/S1600536812036045/tk5143sup1.cif

Structure factors: contains datablock(s) I. DOI: 10.1107/S1600536812036045/tk5143Isup2.hkl

Supplementary material file. DOI: 10.1107/S1600536812036045/tk5143Isup3.cml

Additional supplementary materials:  crystallographic information; 3D view; checkCIF report

## Figures and Tables

**Table 1:** Hydrogen-bond geometry (Å, °) *Cg*2 is the centroid of the C1–C6 phenyl ring.

*D*—H⋯*A*	*D*—H	H⋯*A*	*D*⋯*A*	*D*—H⋯*A*
N1—H1*N*⋯N5^i^	0.84 (2)	2.15 (2)	2.9934 (13)	179 (2)
N3—H3*N*⋯N2^ii^	0.87 (2)	2.09 (2)	2.8947 (13)	154 (2)
N4—H12⋯*Cg*2^iii^	0.85 (2)	2.51 (2)	3.2011 (12)	140 (2)

Symmetry codes: (i) 
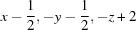
; (ii) 
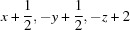
; (iii) 
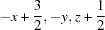
.

**Fig. 1.**
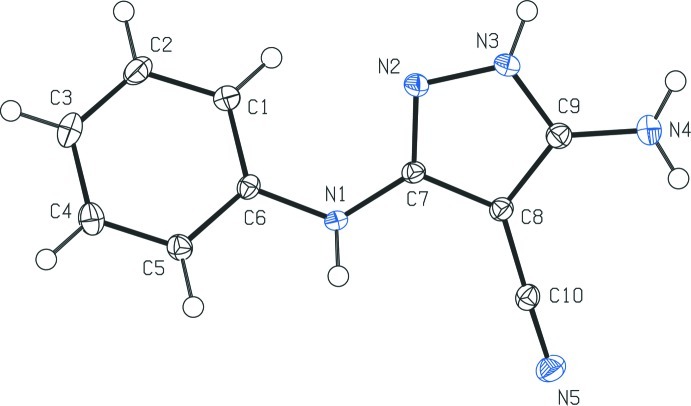
The molecular structure of the title compound with the atom numbering scheme. Displacement ellipsoids for non-H atoms are drawn at the 50% probability level.

**Fig. 2.**
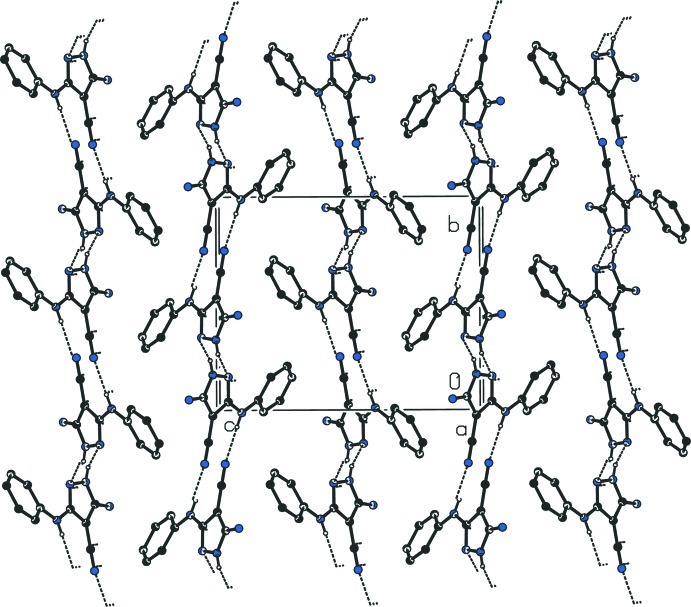
View of chains of the dimers formed by pairs of N—H···N hydrogen bonds, with the R22(12) and R22(6) motifs connected into a supramolecular chain. H atoms not involved in hydrogen bonds have been omitted for clarity.

## supplementary crystallographic information

### Comment

The interest in pyrazole compounds stems from their pharmaceutical and agricultural applications such as drugs, dyes and anaesthetics (Grimmett, 1970; Sheikh *et al.*, 2009; Kaushik *et al.*, 2010; Krishnamurthy *et al.*, 2004). In addition, such pyrazoles and related compounds are common molecules used in coordination or organometallic chemistry as bridging ligands, utilizing the ring positions of the two N atoms (Lynch & McClenaghan, 2005). We report herein the crystal structure of the title compound which was synthesized by our team as a precursor having two functional substituents (amino and nitrile groups) for the purposes of synthesis of multi-fused pyrazolo-heterocyclic compounds such as nitrogen bridgehead derivatives having potential biological activities (Soliman *et al.*, 2010).

In the molecule of the title compound, (Fig. 1), the phenyl and 1*H*-pyrazole ring makes a dihedral angle of 24.00 (6)° with each other.

The crystal structure is stabilized by N—H···N hydrogen bonds (Table 1, Fig. 2) which link the molecules into chains running parallel to [010] with alternating *R*_2_^2^(6) and *R*_2_^2^(12) motifs (Bernstein *et al.*, 1995). In addition, the crystal structure exhibits N—H···π(phenyl) interactions, Table 1, and weak face-to-face π—π stacking interactions [*Cg*1···*Cg*2 (1 + *x*, *y*, *z*) = 3.8890 (6) Å; where *Cg*1 and *Cg*2 are the centroid of the (N2/N3/C7–C9) 1*H*-pyrazole and (C1–C6) phenyl rings].

### Experimental

The title compound was prepared according to the literature procedure (Soliman *et al.*, 2010). Crystals were obtained from an ethanol solution of (I) by slow evaporation (*M*.pt: 481 K).

### Refinement

The hydrogen atoms bound to nitrogen were located from a difference Fourier map and were refined with a distance restraint of N—H = 0.86 (2) Å; their *U*_iso_ values were refined freely. The hydrogen atoms bound to carbon were positioned geometrically and refined using a riding model with C—H = 0.93 Å, and with *U*_iso_ = 1.2*U*_eq_(C). The absolute structure could not be determined reliably; Friedel pairs were not merged.

### Crystal data

**Table:**

C_10_H_9_N_5_	*F*(000) = 416
*M**_r_* = 199.22	*D*_x_ = 1.360 Mg m^−^^3^
Orthorhombic, *P*2_1_2_1_2_1_	Mo *K*α radiation, λ = 0.71073 Å
Hall symbol: P 2ac 2ab	Cell parameters from 9908 reflections
*a* = 6.3441 (1) Å	θ = 2.4–30.5°
*b* = 11.1354 (2) Å	µ = 0.09 mm^−^^1^
*c* = 13.7754 (3) Å	*T* = 90 K
*V* = 973.15 (3) Å^3^	Plate, colourless
*Z* = 4	0.25 × 0.17 × 0.08 mm

### Data collection

**Table:**

Bruker Kappa APEXII DUO diffractometer	2975 independent reflections
Radiation source: fine-focus sealed tube	2767 reflections with *I* > 2σ(*I*)
TRIUMPH curved graphite monochromator	*R*_int_ = 0.035
φ and ω scans	θ_max_ = 30.6°, θ_min_ = 2.4°
Absorption correction: multi-scan (*SADABS*; Sheldrick, 2004)	*h* = −9→9
*T*_min_ = 0.978, *T*_max_ = 0.993	*k* = −15→15
32878 measured reflections	*l* = −19→19

### Refinement

**Table:**

Refinement on *F*^2^	Secondary atom site location: difference Fourier map
Least-squares matrix: full	Hydrogen site location: inferred from neighbouring sites
*R*[*F*^2^ > 2σ(*F*^2^)] = 0.033	H atoms treated by a mixture of independent and constrained refinement
*wR*(*F*^2^) = 0.086	*w* = 1/[σ^2^(*F*_o_^2^) + (0.0494*P*)^2^ + 0.1533*P*] where *P* = (*F*_o_^2^ + 2*F*_c_^2^)/3
*S* = 1.09	(Δ/σ)_max_ = 0.001
2975 reflections	Δρ_max_ = 0.32 e Å^−^^3^
152 parameters	Δρ_min_ = −0.20 e Å^−^^3^
4 restraints	Absolute structure: nd
Primary atom site location: structure-invariant direct methods	

### Special details

**Table:**

Geometry. Bond distances, angles *etc*. have been calculated using the rounded fractional coordinates. All su's are estimated from the variances of the (full) variance-covariance matrix. The cell e.s.d.'s are taken into account in the estimation of distances, angles and torsion angles
Refinement. Refinement on *F*^2^ for ALL reflections except those flagged by the user for potential systematic errors. Weighted *R*-factors *wR* and all goodnesses of fit *S* are based on *F*^2^, conventional *R*-factors *R* are based on *F*, with *F* set to zero for negative *F*^2^. The observed criterion of *F*^2^ > σ(*F*^2^) is used only for calculating -*R*-factor-obs *etc*. and is not relevant to the choice of reflections for refinement. *R*-factors based on *F*^2^ are statistically about twice as large as those based on *F*, and *R*-factors based on ALL data will be even larger.

### Fractional atomic coordinates and isotropic or equivalent isotropic displacement parameters (Å^2^)

**Table:**

	*x*	*y*	*z*	*U*_iso_*/*U*_eq_	
N1	0.50345 (15)	−0.01004 (8)	0.89839 (7)	0.0142 (2)	
N2	0.71282 (15)	0.15769 (8)	0.94613 (7)	0.0154 (2)	
N3	0.89266 (15)	0.16812 (8)	1.00299 (7)	0.0168 (2)	
N4	1.13973 (17)	0.05235 (10)	1.09010 (8)	0.0234 (3)	
N5	0.84875 (15)	−0.24944 (9)	1.03572 (7)	0.0204 (3)	
C1	0.40580 (18)	0.15607 (10)	0.78868 (8)	0.0173 (3)	
C2	0.2548 (2)	0.20761 (11)	0.72811 (8)	0.0205 (3)	
C3	0.06057 (19)	0.15345 (11)	0.71341 (8)	0.0213 (3)	
C4	0.01783 (19)	0.04497 (11)	0.75945 (8)	0.0193 (3)	
C5	0.16597 (18)	−0.00746 (10)	0.82040 (7)	0.0152 (3)	
C6	0.36064 (16)	0.04854 (9)	0.83683 (7)	0.0130 (2)	
C7	0.66942 (16)	0.04110 (9)	0.94711 (7)	0.0122 (2)	
C8	0.81769 (17)	−0.02408 (9)	1.00460 (8)	0.0133 (2)	
C9	0.96026 (17)	0.06332 (10)	1.03764 (8)	0.0153 (3)	
C10	0.83277 (17)	−0.14847 (9)	1.02122 (7)	0.0145 (2)	
H1	0.53600	0.19300	0.79700	0.0210*	
H1N	0.459 (3)	−0.0771 (13)	0.9176 (12)	0.027 (4)*	
H2	0.28480	0.27970	0.69700	0.0250*	
H3	−0.03960	0.18910	0.67340	0.0260*	
H3N	0.958 (3)	0.2358 (13)	1.0116 (14)	0.034 (5)*	
H4	−0.11110	0.00720	0.74930	0.0230*	
H5	0.13580	−0.08020	0.85050	0.0180*	
H11	1.200 (3)	0.1170 (14)	1.1129 (13)	0.038 (5)*	
H12	1.165 (3)	−0.0151 (14)	1.1162 (13)	0.036 (5)*	

### Atomic displacement parameters (Å^2^)

**Table:**

	*U*^11^	*U*^22^	*U*^33^	*U*^12^	*U*^13^	*U*^23^
N1	0.0145 (4)	0.0093 (4)	0.0189 (4)	−0.0012 (3)	−0.0034 (4)	0.0031 (3)
N2	0.0152 (4)	0.0112 (4)	0.0197 (4)	−0.0003 (3)	−0.0030 (3)	−0.0012 (3)
N3	0.0178 (4)	0.0117 (4)	0.0208 (4)	−0.0023 (3)	−0.0038 (3)	−0.0005 (3)
N4	0.0219 (5)	0.0230 (5)	0.0253 (5)	−0.0035 (4)	−0.0095 (4)	0.0041 (4)
N5	0.0189 (5)	0.0160 (4)	0.0262 (5)	0.0023 (4)	0.0001 (4)	0.0038 (4)
C1	0.0189 (5)	0.0150 (5)	0.0179 (5)	−0.0012 (4)	−0.0018 (4)	0.0018 (4)
C2	0.0260 (6)	0.0172 (5)	0.0183 (5)	0.0030 (4)	−0.0029 (4)	0.0044 (4)
C3	0.0206 (5)	0.0250 (6)	0.0182 (5)	0.0068 (5)	−0.0037 (4)	0.0018 (4)
C4	0.0144 (5)	0.0251 (5)	0.0184 (5)	0.0007 (4)	−0.0004 (4)	−0.0010 (4)
C5	0.0134 (5)	0.0170 (4)	0.0153 (4)	−0.0011 (4)	0.0010 (4)	0.0006 (4)
C6	0.0136 (4)	0.0126 (4)	0.0129 (4)	0.0025 (4)	−0.0004 (3)	−0.0002 (3)
C7	0.0124 (4)	0.0105 (4)	0.0137 (4)	0.0007 (4)	0.0005 (4)	0.0003 (3)
C8	0.0128 (4)	0.0126 (4)	0.0145 (4)	0.0008 (3)	0.0001 (4)	0.0011 (3)
C9	0.0157 (4)	0.0155 (5)	0.0147 (4)	−0.0005 (4)	0.0002 (3)	0.0004 (4)
C10	0.0125 (4)	0.0164 (4)	0.0147 (4)	0.0011 (4)	0.0008 (3)	0.0017 (3)

### Geometric parameters (Å, º)

**Table:**

N1—C6	1.4020 (14)	C2—C3	1.3868 (17)
N1—C7	1.3724 (14)	C3—C4	1.3910 (17)
N2—N3	1.3888 (14)	C4—C5	1.3889 (16)
N2—C7	1.3272 (13)	C5—C6	1.4019 (15)
N3—C9	1.3318 (14)	C7—C8	1.4279 (15)
N4—C9	1.3541 (15)	C8—C9	1.4044 (15)
N5—C10	1.1464 (14)	C8—C10	1.4072 (14)
N1—H1N	0.841 (15)	C1—H1	0.9300
N3—H3N	0.868 (16)	C2—H2	0.9300
N4—H11	0.874 (17)	C3—H3	0.9300
N4—H12	0.848 (16)	C4—H4	0.9300
C1—C2	1.3940 (16)	C5—H5	0.9300
C1—C6	1.3985 (15)		
			
C6—N1—C7	126.77 (9)	N1—C7—N2	124.06 (9)
N3—N2—C7	104.27 (8)	N1—C7—C8	124.46 (9)
N2—N3—C9	113.17 (9)	C7—C8—C9	104.58 (9)
C6—N1—H1N	112.8 (13)	C7—C8—C10	129.45 (10)
C7—N1—H1N	118.2 (12)	C9—C8—C10	125.85 (10)
N2—N3—H3N	122.8 (12)	N3—C9—N4	122.76 (10)
C9—N3—H3N	123.9 (12)	N3—C9—C8	106.48 (9)
C9—N4—H12	117.7 (12)	N4—C9—C8	130.69 (11)
H11—N4—H12	119.7 (17)	N5—C10—C8	178.64 (11)
C9—N4—H11	119.1 (12)	C2—C1—H1	120.00
C2—C1—C6	119.71 (10)	C6—C1—H1	120.00
C1—C2—C3	121.26 (11)	C1—C2—H2	119.00
C2—C3—C4	118.97 (11)	C3—C2—H2	119.00
C3—C4—C5	120.58 (11)	C2—C3—H3	121.00
C4—C5—C6	120.45 (10)	C4—C3—H3	121.00
N1—C6—C1	123.57 (9)	C3—C4—H4	120.00
N1—C6—C5	117.39 (9)	C5—C4—H4	120.00
C1—C6—C5	119.00 (9)	C4—C5—H5	120.00
N2—C7—C8	111.48 (9)	C6—C5—H5	120.00
			
C7—N1—C6—C5	−159.01 (10)	C2—C3—C4—C5	0.99 (17)
C6—N1—C7—N2	3.39 (17)	C3—C4—C5—C6	0.32 (16)
C7—N1—C6—C1	23.65 (16)	C4—C5—C6—C1	−1.90 (15)
C6—N1—C7—C8	−176.20 (10)	C4—C5—C6—N1	−179.36 (10)
C7—N2—N3—C9	0.38 (12)	N1—C7—C8—C9	178.44 (10)
N3—N2—C7—C8	0.53 (12)	N2—C7—C8—C10	−177.26 (11)
N3—N2—C7—N1	−179.10 (10)	N1—C7—C8—C10	2.37 (18)
N2—N3—C9—N4	176.12 (10)	N2—C7—C8—C9	−1.19 (12)
N2—N3—C9—C8	−1.13 (13)	C7—C8—C9—N3	1.35 (12)
C6—C1—C2—C3	−0.88 (17)	C10—C8—C9—N4	0.7 (2)
C2—C1—C6—N1	179.46 (10)	C7—C8—C9—N4	−175.60 (12)
C2—C1—C6—C5	2.16 (16)	C10—C8—C9—N3	177.60 (10)
C1—C2—C3—C4	−0.71 (17)		

### Hydrogen-bond geometry (Å, º)

Cg2 is the centroid of the C1–C6 phenyl ring.

**Table:**

*D*—H···*A*	*D*—H	H···*A*	*D*···*A*	*D*—H···*A*
N1—H1*N*···N5^i^	0.84 (2)	2.15 (2)	2.9934 (13)	179 (2)
N3—H3*N*···N2^ii^	0.87 (2)	2.09 (2)	2.8947 (13)	154 (2)
C1—H1···N2	0.93	2.37	2.9152 (15)	117
N4—H12···*Cg*2^iii^	0.85 (2)	2.51 (2)	3.2011 (12)	140 (2)

Symmetry codes: (i) *x*−1/2, −*y*−1/2, −*z*+2; (ii) *x*+1/2, −*y*+1/2, −*z*+2; (iii) −*x*+3/2, −*y*, *z*+1/2.
